# DNA methylation alterations in iPSC- and hESC-derived neurons: potential implications for neurological disease modeling

**DOI:** 10.1186/s13148-018-0440-0

**Published:** 2018-01-29

**Authors:** Laura de Boni, Gilles Gasparoni, Carolin Haubenreich, Sascha Tierling, Ina Schmitt, Michael Peitz, Philipp Koch, Jörn Walter, Ullrich Wüllner, Oliver Brüstle

**Affiliations:** 1 Department of Neurology, University Hospital of Bonn, Bonn, Germany; 2 Institute of Reconstructive Neurobiology, Life & Brain Center, University of Bonn, Bonn, Germany; 3 Institute for Genetics/Epigenetics, FR8.3 Life Sciences, Saarland University, Saarbrücken, Germany; 4 German Center for Neurodegenerative Diseases (DZNE), University of Bonn, Bonn, Germany

**Keywords:** DNA methylation, Isogenic stem cells, iPS cell-derived neurons

## Abstract

**Background:**

Genetic predisposition and epigenetic alterations are both considered to contribute to sporadic neurodegenerative diseases (NDDs) such as Parkinson’s disease (PD). Since cell reprogramming and the generation of induced pluripotent stem cells (iPSCs) are themselves associated with major epigenetic remodeling, it remains unclear to what extent iPSC-derived neurons lend themselves to model epigenetic disease-associated changes. A key question to be addressed in this context is whether iPSC-derived neurons exhibit epigenetic signatures typically observed in neurons derived from non-reprogrammed human embryonic stem cells (hESCs).

**Results:**

Here, we compare mature neurons derived from hESC and isogenic human iPSC generated from hESC-derived neural stem cells. Genome-wide 450 K-based DNA methylation and HT12v4 gene array expression analyses were complemented by a deep analysis of selected genes known to be involved in NDD. Our studies show that DNA methylation and gene expression patterns of isogenic hESC- and iPSC-derived neurons are markedly preserved on a genome-wide and single gene level.

**Conclusions:**

Overall, iPSC-derived neurons exhibit similar DNA methylation patterns compared to isogenic hESC-derived neurons. Further studies will be required to explore whether the epigenetic patterns observed in iPSC-derived neurons correspond to those detectable in native brain neurons.

**Electronic supplementary material:**

The online version of this article (10.1186/s13148-018-0440-0) contains supplementary material, which is available to authorized users.

## Background

The origin and pathophysiology of sporadic neurodegenerative diseases (NDDs) such as idiopathic Parkinson’s disease (iPD) still remain enigmatic. In this regard, epigenetic alterations of genes including α-synuclein (*SNCA*) could contribute to the pathophysiology of this devastating disorder or the individual susceptibility [1].

Previously, we have identified significantly reduced DNA methylation levels of *SNCA* intron 1 in PD brains [2]. In SK-N-SH cells, DNA demethylation of the *SNCA* intron 1—using the DNA methyltransferase inhibitor 5-Azacytidine—was associated with an increase in *SNCA* mRNA and α-synuclein protein expression [2]. This is an important finding as elevated α-synuclein protein levels are directly linked to the development of PD [3, 4]. To date, these findings could not be verified in follow-up studies [5] and/or in native neurons derived from iPD patients compared to controls due to the inaccessibility of living disease-affected cells.

In this context, in vitro differentiated neural cells from patient-derived induced pluripotent stem cells (iPSCs) could provide a tool to study human neurons. Human iPSCs have indeed already been employed to dissect the mechanisms of several mostly inherited NDDs [6–9]. iPSC-based models of PD, mainly focusing on familial forms of the disease, displayed some interesting and relevant phenotypes [10]. Consequently, patient-specific iPSC-derived neurons could be used to establish in vitro models for iPD allowing epigenetic and gene expression studies. Recently, a comprehensive study comprising epigenetic analyses in PD iPSC models revealed an aberrant epigenome in iPSC-derived dopaminergic neurons (DAn) of iPD and LRRK2 PD patients [11]. These data suggest that cell-specific and presumptive disease-associated epigenetic changes can be retrieved from iPSC-derived patient-specific neurons. Nevertheless, the process of induced reprogramming is based on erasing the existing epigenetic state of the cell of origin [12, 13]. One key question emerging in the context of pluripotent stem cell-based disease modeling is whether iPSC-derived neurons display epigenetic patterns resembling those of neurons derived from non-reprogrammed human embryonic stem cells (hESCs). To address this question, we employed an isogenic stem cell system to compare DNA methylation patterns of hESC- and iPSC-derived mature neurons both generated from the same parental hESC line. Such an isogenic approach should eliminate differences due to individual genetic variation. Specifically, we assessed (i) genome-wide DNA methylation of > 485.000 CpGs in potential regulatory elements such as promoters or enhancers, (ii) gene expression of > 47.000 transcripts, and (iii) DNA methylation and gene expression changes of several NDD-related genes, in particular, *SNCA*.

## Results

### Genome-wide and single gene DNA methylation patterns of isogenic hESC- and iPSC-derived neurons are very similar

**Fig. 1:**
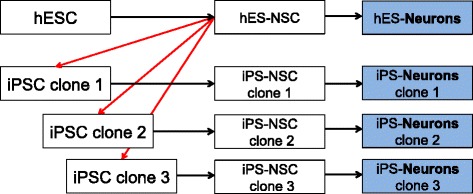
Isogenic human stem cell system. Stable populations of human embryonic stem cell (hESC)-derived neural stem cells (hES-NSC, female hESC line I3) were reprogrammed to pluripotency, and three iPSC clones were subsequently re-differentiated into neural stem cells (NSCs) and neurons

The current study addressed the question whether iPSC-derived neurons display similar DNA methylation patterns compared to hESC-derived neurons. As a first step, we compared isogenic hESC- and iPSC-derived neurons, which allow the analysis of DNA methylation differences independently of individual genetic variations. To that end, we already differentiated thoroughly the characterized and stable populations of hESC-derived stable neural stem cells (NSCs) from the female hESC line I3 into 6-week-old neurons (Fig. 1). These hESC-derived NSCs, also referred to as long-term self-renewing neuroepithelial stem cells (lt-NES cells) differentiate predominantly into GABAergic neurons with a posterior identity corresponding to the ventral hindbrain area [14]. We reprogrammed these NSCs to iPSC and subsequently differentiated them into three clonal populations of iPSC-derived neurons, which were compared with neurons generated from the parental hESC (Fig. 1). A genome-wide analysis was carried out using Illumina 450 K bead arrays measuring DNA methylation levels at > 450.000 CpG sites covering promoters and putative regulatory domains of all designable RefSeq genes.

**Fig. 2:**
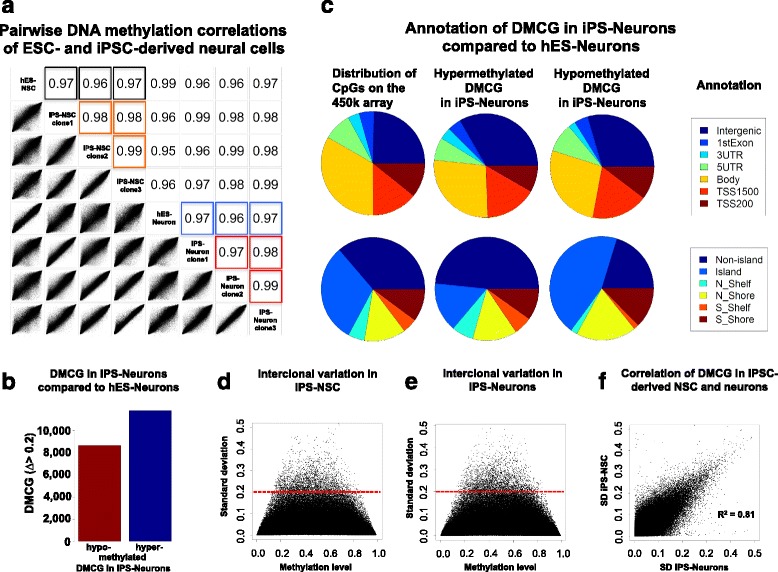
Genome-wide DNA methylation analysis. **a** Pairwise correlation plots (Pearson correlation, genome-wide DNA methylation analysis) of hESC- and iPSC-derived NSC and neurons display high correlation coefficients (black: hES-NSC vs. iPS-NSC, blue: hES-Neurons vs. iPS-Neurons) and minimal interclonal variance (orange: comparison of iPS-NSC clone 1, clone 2, and clone 3; red: comparison of iPS-Neurons clone 1, clone 2, and clone 3). **b** Number of differentially hypo- and hypermethylated CpGs (DMCG) in iPS-Neurons compared to hES-Neurons. iPSC-derived neurons demonstrate slightly more hyper- than hypomethylated DMCG compared to hESC-derived neurons. **c** Annotation of hypo- and hypermethylated DMCG in terms of gene regulatory regions (intergenic gene regions, 1st exon, 3′ and 5′ untranslated region (UTR), gene body, promoter areas: transcription start sites (TSS) 1500 and 200; upper pie charts), and CpG islands (CpG islands and flanking regions before (N_Shelf, N_Shore) and after (S_Shelf, S_Shore) CpG islands; lower pie charts) in comparison to the overall distribution of markers on the whole 450 K array (left pie charts), respectively. **d**, **e** Interclonal variation of DNA methylation is pronounced at CpGs with intermediate methylation levels in iPS-Neurons (**d**) and -NSC (**e**), respectively. **f** Variation of DMCG is highly correlated between iPS-NSC and iPS-Neurons. SD, standard deviation

A Pearson correlation analysis of gene-linked CpG methylation revealed at least a 96% concordance of iPS-NSC and iPS-Neurons compared to their hESC-derived counterparts (Fig. 2a). The interclonal variance was small; NSC and neurons derived from the three different iPSC clones showed a concordance for DNA methylation levels of at least 97% (Fig. 2a).

Differentially methylated CpGs (DMCGs) in iPSC-derived neurons compared to their hESC-derived counterparts (defined by a methylation difference > 20%) were analyzed in more detail; 8.680 (42.35%) of these DMCGs were hypomethylated, and 11.816 (57.65%) were hypermethylated in iPS- compared to hES-Neurons (Fig. 2b). Transcription start sites were not overrepresented in these DMCGs compared to the overall 450 K array annotation (Fig. 2c upper panels). More hypomethylated CpGs were found in CpG islands (CGI) and more hypermethylated CpGs in non-CGI DNA sequences (Fig. 2c lower panels). We also analyzed the standard deviation (> 0.2) of single DMCG in iPS-NSC (Fig. 2d) and iPS-Neurons (Fig. 2e) to identify an interclonal variation of DMCG. We identified only 3.051 and 3.264 interclonal DMCG sites with a standard deviation of > 0.2 for iPS-NSC and iPS-Neurons, respectively. We found a high correlation of variation between iPSC-derived NSC and neurons (Fig. 2d–f). To identify gene loci showing the most prominent alterations, we carried out an interindividual analysis (hES-Neurons vs. each iPSC-derived neuronal clone) applying a more stringent threshold of > 0.5 methylation difference (Additional file 1 A–C). In line with the previous results, we could identify only a few DMCGs (*n* = 1010 up to *n* = 1802) being differentially methylated comparing hES-Neurons to all three iPSC-derived neuronal clones (Additional file 1 A–C). The data indicate that (i) different iPSC neuronal clones exhibit almost the same amount of DMCG when compared to their hESC counterparts and (ii) that the differences are not biased toward certain genomic sites, keeping in mind that the distribution of CpGs on the 450 K array across the genome is not equally distributed per se. Using Fisher’s test, we could, however, identify a highly significant variation for methylation differences on the X chromosome in general (Additional file 1 D).

### Consistent gene expression patterns of isogenic hESC- and iPSC-derived neurons

To compare gene expression patterns of hESC- and iPSC-derived neurons, we performed HT12v4 array experiments covering more than 47.000 transcripts. We tested the technical variation of the assay by hybridizing all the neuronal samples in duplicates. We observed high fidelity illustrated by correlation values of greater than 98% between each pair of replicates and a high interclonal correlation of hESC- and iPSC-derived neurons (Additional file 2 A–D). For all downstream comparisons, replicate 1 of each sample was used.

**Fig. 3:**
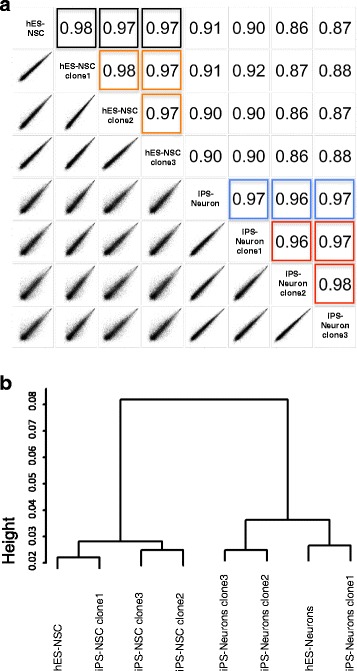
Genome-wide expression analysis. **a** Pairwise correlation plots (Pearson correlation, genome-wide expression analysis) of hESC- and iPSC-derived NSC and neurons display high correlation coefficients (black: hES-NSC vs. iPS-NSC, blue: hES-Neurons vs. iPS-Neurons) and minimal interclonal variance (orange: comparison of iPS-NSC clone 1, clone 2, and clone 3; red: comparison of iPS-Neurons clone 1, clone 2, and clone 3). **b** Hierarchical analysis demonstrates clustering of iPS-Neurons and hES-Neurons and clustering of iPS-NSC together with hES-NSC. Height 0.02 displays a similarity of 98% in gene expression levels

Pearson correlation analysis revealed at least a 96% concordance of gene expression profiles of iPSC- and hESC-derived neurons; interclonal variance was minimal (< 4%, Fig. 3a, Additional file 2 E, F). These data revealed that neurons generated from iPSC are very similar to their hESC-derived counterparts at the gene expression level. Hierarchical cluster analysis revealed that hESC-derived and iPSC-derived neurons cluster together, as do their parental hESC-derived and iPSC-derived NSC. These data indicate that differentiation-associated expression changes outweigh potential changes due to the reprogramming procedure (Fig. 3b). Differentially expressed genes (DEG) in iPS-Neurons were mostly associated with transcription regulation and homeobox genes. Overall, the coefficient of determination for gene expression levels in iPS-NSC and iPS-Neurons amounted to *R*^2^ = 0.75 (data not shown).

**Fig. 4:**
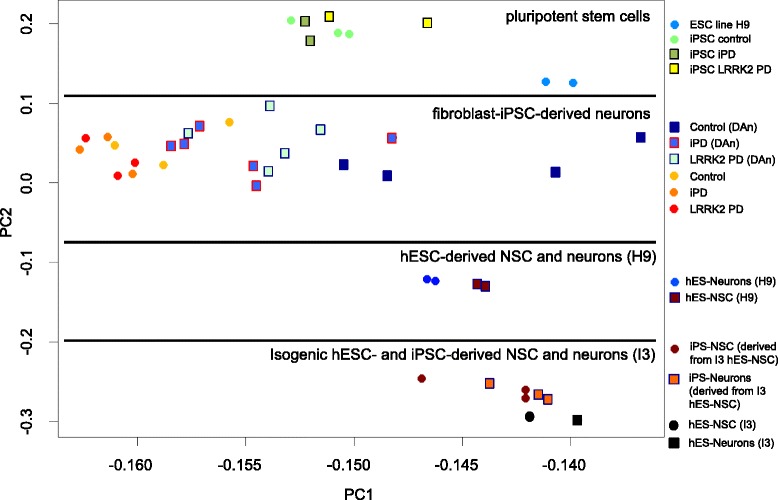
Comparative analysis of DNA methylation in pluripotent cells and neural derivatives. In silico PCA of 450 K-based DNA methylation data from pluripotent stem cells and neural cells generated from fibroblast-derived iPSC [11] and H9 hESC [15] as well as isogenic I3 hESC- and iPSC. Fibroblast-derived iPSC originate from iPD and LRRK2 patients as well as healthy control donors

Taken together, our data indicate that isogenic hESC-derived and iPSC-derived neurons generated via a highly standardized intermediate NSC population [14] display similar DNA methylation and gene expression patterns. Thus, the reprogramming process does not result in major alterations of the methylation and gene expression patterns in iPSC-derived neurons. It is important to note though that the iPSCs in our experimental setting were generated from hESC-derived NSC, while patient-derived iPSCs are typically generated from fibroblasts or peripheral blood mononuclear cells. To assess how methylation levels observed in our system relate to the data obtained from neurons generated from fibroblast-derived iPSC, we performed a comparative in silico analysis including hESC-derived neural cells from another hESC line (H9) and fibroblast-derived neuronal populations from controls, iPD and LRRK2 PD patients [11, 15]. Hypothesis-free variant principal component analysis (PCA) of the 450 K data revealed that pluripotent stem cells, fibroblast-iPSC-derived neurons, H9 hESC-derived neural cells, and I3 hESC-derived neural cells clustered in distinct tiers (Fig. 4 and Additional file 3). PC2 was most likely associated with cell type, whereas the biggest variance (PC1) could not be attributed to a single factor. The segregation pattern of the different neuronal populations suggests that the individual genetic background (patient-derived vs. H9 ESC-derived vs. I3 ESC-derived) and differences in the neuronal differentiation protocols have a much higher impact on the methylation landscape than the reprogramming process itself. In line with this notion, all isogenic neurons generated from I3 ESC and I3 ESC-NSC-derived iPSC formed a tight cluster, which segregated from fibroblast- and H9 ESC-derived neurons (Fig. 4). We could also identify a weak, but significant correlation regarding PC1 and batch effects (*p* < 0.0002, *R*^2^ = 0.73). From our observations, we conclude that a thorough comparison of neurons generated from ESC-NSC- and fibroblast-derived iPSC would require isogenic human ESC and iPSC. Such comparative studies might be extended to iPSC-derived vs. central nervous system (CNS) tissue-derived neurons isolated from the same donor, which could in the end resolve to what extent the epigenetic landscape of reprogrammed neurons reflects that of primary human neurons.

### DNA methylation changes during differentiation of isogenic hESC- and iPSC-derived neurons

**Fig. 5:**
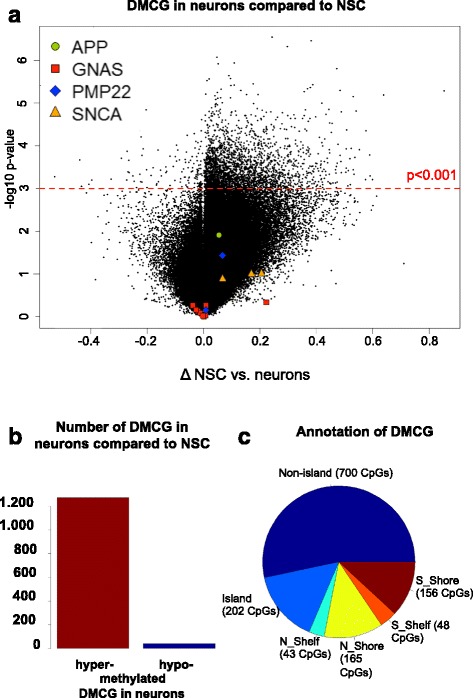
Genome-wide and single gene DNA methylation analysis. **a** Volcano plot showing CpG methylation changes during neuronal differentiation. HESC- and iPSC-derived NSC and neurons were grouped together due to their high similarity, respectively. We calculated the difference for each NSC and neuronal pair (Δ) based on a paired *t* test of *p* < 0.001 (red line). Δ of 0.2 represents 20% difference in CpG methylation. We detected minor genome-wide DMCG. Neurons exhibited more significantly hypermethylated CpGs compared to their matched NSC (positive values) than hypomethylated CpGs (negative values). Significant DNA methylation changes during neuronal differentiation of selected NDD-associated single genes (*APP*, *PMP22*, *SNCA*, *GNAS*) were not detected. **b** Number of differentially hypo- and hypermethylated CpGs (DMCG) in hESC- and iPSC-derived neurons compared to NSC, respectively. Neurons demonstrate more hyper- than hypomethylated DMCG compared to NSC. **c** Annotation of hypo- and hypermethylated DMCG at different gene regulatory regions (CpG islands) in hESC- and iPSC-derived neurons compared to NSC

To assess the acquisition of methylation patterns during neuronal differentiation in depth, we pooled the data of the hESC- and iPSC-derived NSC and compared them to the pooled data of hESC- and iPSC-derived neurons. We performed a paired *t* test (*p* < 0.001) applying a less conservative methylation difference of 10%, which identified 1.314 DMCG only (Fig. 5a, Additional file 4). Only 41 CpGs were hypomethylated, while the vast majority (1.273 CpGs) was hypermethylated in neurons compared to NSC (Fig. 5b). We did not identify an obvious enrichment for DMCG comparing gene sub-regions such as transcription start sites, promoter, and gene body regions in hES- and iPS-Neurons compared to NSC (Additional file 5), but less DMCG in CpG island regions compared to other annotations such as CpG shelfs or shores (Fig. 5c).

### DNA methylation levels of SNCA intron 1 increased during neuronal differentiation in isogenic hESC- and iPSC-derived neurons

**Fig. 6:**
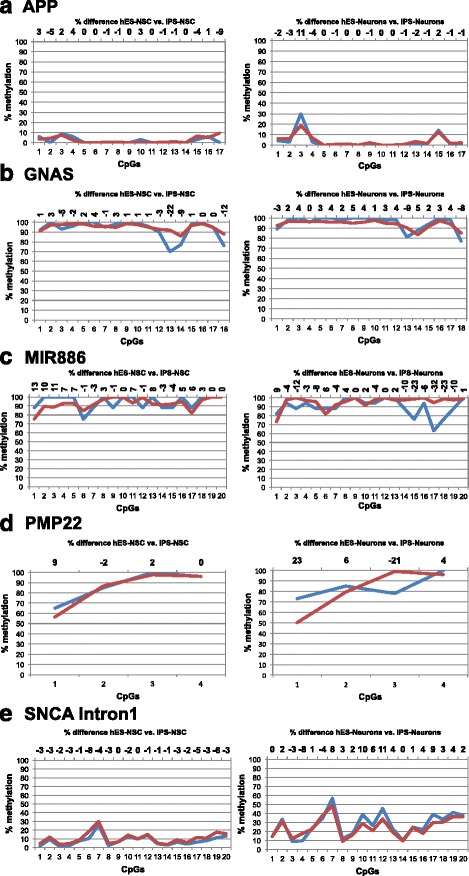
Single gene DNA methylation analysis. **a**-**e** DNA methylation levels (%) of individual CpGs of single genes from isogenic hESC-derived and iPSC-derived NSC and neurons (*APP*, *GNAS*, *MIR886*, *PMP22*, *SNCA* intron 1). The % difference at each CpG between hESC-derived and iPSC-derived neurons is depicted at the secondary x-axis. Neurons were differentiated for 6 weeks. Blue = hESC-derived NSC or neurons; red = iPSC-derived NSC or neurons

We next used MiSeq amplicon sequencing of bisulphite-treated genomic DNA (Bi-PROF; [16]) to validate our data obtained from the 450 K assay and to specifically assess DNA methylation at a single base level. We choose dosage-sensitive genes known to be involved in different neurological disorders, including *APP* (gene duplication, Alzheimer’s disease, AD), *SNCA* (gene duplication/triplication, Parkinson’s disease, PD) [17], and *PMP22*, which has been associated with Charcot-Marie-Tooth 1A (gene duplication) and several other CNS disorders [18, 19]. Epigenetic modification of such dosage-sensitive genes could represent a missing link between familial and sporadic forms of NDD [1]. Furthermore, we chose MIR886, which was found to be differentially methylated in PD [20] and *GNAS*, a gene with a highly complex imprinted expression pattern [21] (Additional file 6). All aforementioned samples (hES-Neuron and iPS-Neuron clones 1–3) were included in the analysis. Pearson correlation coefficients demonstrated a correlation of 0.92 for individual CpGs detected by 450 K bead array and Bi-PROF, respectively. After a successful validation of single CpGs obtained from the 450 K assay, we analyzed amplicons located at the promoter regions and CpG islands of the candidate genes (Additional file 6). A comparison of hESC- and iPSC-derived neurons revealed that most regulatory regions exhibited unchanged DNA methylation patterns; the DNA methylation levels of *APP*, *GNAS*, *MIR886*, and *SNCA* promoter and intron 1 were similar comparing hESC- and iPSC-derived neurons (Fig. 6, Additional file 7). Average methylation levels over all CpGs were also comparable between hESC- and iPSC-derived NSC (Fig. 6, Additional file 7). The pattern within the *SNCA* intron 1 displayed higher mean methylation levels over all analyzed CpGs compared to the canonical promoter region in line with the previous analysis of bulk native brain tissue (Additional file 7) [2, 5]. These data show that DNA methylation levels at promoter regions of the selected candidate genes are similar in ESC- and iPSC-derived neurons.

**Fig. 7:**
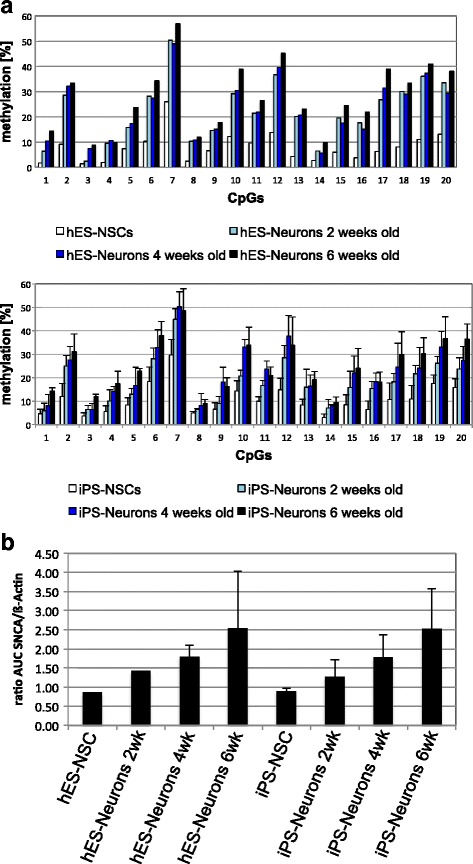
DNA methylation and gene expression patterns of *SNCA* during neuronal differentiation. **a** DNA methylation levels of *SNCA* intron 1 in hESC- (*n* = 1) and iPSC-derived neural cells (*n* = 3). **b** Semiquantitative PCR of *SNCA* mRNA levels in hESC- (*n* = 1) and iPSC-derived neural cells (*n* = 3). Wk, weeks; AUC, area under the concentration-time curve

DNA methylation alterations during neuronal differentiation of NSCs were observed specifically at the *SNCA* intron 1, but not for the other selected and analyzed NDD-associated genes. HES-NSC generated from I3 hESC and iPS-NSC generated from I3 hES-NSC-derived iPSC exhibited slightly higher, though non-significant (iPSC vs. iPS-NSC *p* = 0.08) DNA methylation levels of *SNCA* intron 1 than their parental PSC populations and hESC of the line I6; differentiation of the iPSC-derived NSC line I3 resulted in a further significant increase in DNA methylation (ESC vs. iPS-Neurons *p* = 0.03, iPSC vs. iPS-Neurons *p* = 0.02, iPS-NSC vs. iPS-Neurons *p* = 0.02; Additional file 8). To obtain more insight into DNA methylation changes in the *SNCA* intron 1 across neuronal differentiation, we analyzed NSC and neurons differentiated for 2, 4, and 6 weeks (Fig. 7a). Mean DNA methylation levels over all analyzed CpG sites of *SNCA* intron 1 increased during a 6-week period of neuronal differentiation in both hESC-derived (2.3-fold) and iPSC-derived neurons (3.5-fold, *p* = 0.016; Fig. 7a). While DNA methylation levels increased at each CpG upon neuronal differentiation, CpGs 3, 5, and 20 showed prominent changes (*p* = 0.001, *p* = 0.002, and *p* = 0.008, respectively; Fig. 7a). Remarkably, even at this single CpG level, methylation changes in iPSC- and hESC-derived neurons were very similar and showed a comparable pattern (Fig. 7a). Interestingly, increasing DNA methylation of *SNCA* intron 1 across neuronal differentiation was not associated with decreased but increased *SNCA* expression in both hESC- and iPSC-derived neurons (Fig. 7b).

## Discussion

### Impact of epigenetic alterations in NDD

There is growing evidence that epigenetic alterations might play a role in NDD [1, 22]. In particular, epigenetic modifications of dosage-sensitive genes such as *SNCA*, a key player in PD, could be important for the phenotypic features, individual susceptibility, and the variable course of NDD [17]. However, the exact impact of epigenetic modifications in NDD is still unknown. To date, epigenetic studies have been hampered by the inaccessibility of disease-affected cells. Previous studies on bulk brain tissue aimed at exploring whether DNA methylation changes of the *SNCA* gene could contribute to the development of iPD [2, 5], but the results were not conclusive. iPSC-derived neurons could represent an attractive experimental tool to study epigenetic alterations in PD [23]. However, the reprogramming process itself is based on a major epigenetic “reset” with the resulting iPSC exhibiting epigenetic patterns closely related to those of ESC [24, 25]. This is presumably associated with a loss of both, epigenetic somatic memory (i.e., tissue-specific epigenetic patterns of the donor cells used for iPSC generation) and age-associated epigenetic signatures [26–28]. The latter is particularly relevant as NDD typically occur in late age. A global epigenetic rearrangement during the generation of iPSC could largely eliminate epigenetic alterations resulting from prior noxious exposures. IPSC might thus not fully reflect *acquired* patient-specific epigenetic changes. On the other hand, epigenetic alterations are not only due to environmental impacts but also reflect changes *in cis*, i.e., changes in methylation patterns as a downstream result of genetic variants [29].

### Isogenic stem cell systems

It is evident that successful and reliable in vitro modeling of epigenetic changes depends on the faithful replication of the epigenetic signatures observed in non-reprogrammed cells. The comparative assessment of epigenetic patterns in reprogrammed versus non-reprogrammed cells is impeded by the interindividual genetic variability. Isogenic systems can be used to resolve this problem. Indeed, while this manuscript was in preparation, several studies started to address the issue of reprogramming-associated epigenetic differences and their biological significance in isogenic stem cell systems [24, 30, 31]. Teichroeb et al. compared the transcriptome of isogenic hESC (male hESC line H9) and iPSC using an array-based analysis (HT-12v3) [30]. They found a striking similarity (99.6%) of the transcriptome of isogenic hESC and iPSC [30]. These results were supported by Mallon et al. who compared array-based DNA methylation (Illumina Meth.27) and gene expression (Agilent 4x44k One Color) patterns of isogenic hESC (male ESC-line H1) and iPSC [31]. They found no major differences in global gene expression patterns and an accordance of DNA methylation patterns of 98–99% [31]. Choi et al. compared isogenic iPSC and hESC derived from two male hESC lines (HUES2 and HUES3) [24]. Employing global expression profiling by RNA sequencing, they found that only 49 genes were differentially expressed in these isogenic cell populations [24]. DNA methylation pattern differences (reduced representation bisulphite sequencing, RRBS) accounted for only approximately 4% [24].

We were interested in extending these comparative studies from pluripotent stem cells to their neuronal derivatives. To that end, we compared DNA methylation patterns of reprogrammed and non-reprogrammed mature neurons derived from the same hESC line. Employing an isogenic human neural stem cell system, we found that the genome-wide DNA methylations (and gene expression profiles) in hESC- and iPSC-derived neurons are remarkably similar. Furthermore, iPSC-derived cells displayed only minor interclonal variability. These results are in line with several studies on epigenetic and gene expression patterns in isogenic pluripotent stem cell populations [24, 30, 31]. The need for an isogenic system is also underlined by the results of our comparative in silico analysis including hESC-derived neural cells from another hESC line (H9) and fibroblast-derived neuronal populations from controls, iPD, and LRRK2 PD patients [11, 15]. These data indicate that the individual genetic background (patient-derived vs. H9 ESC-derived vs. I3 ESC-derived) and differences in the neuronal differentiation protocols have a much higher impact on the methylation landscape than the reprogramming process itself.

Looking at five NDD-related candidate genes within our isogenic system, we also observed a high similarity between hESC- and iPSC-derived neural cell methylation patterns at the single base level. Furthermore, we detected a high DNA methylation similarity of hESC- and iPSC-derived neurons concerning the imprinted locus *GNAS*. Interestingly, we found a distinct increase of DNA methylation in *SNCA* intron 1 upon neuronal differentiation up to 6 weeks in vitro. ESC- and iPSC-derived neurons acquired a highly comparable pattern during neuronal differentiation. However, this differentiation-associated increase in methylation was paralleled by increased expression of *SNCA*. It remains currently unclear whether DNA methylation changes upon neuronal differentiation of iPSC- and hESC-derived neurons indeed influence *SNCA* gene expression levels.

### Limitations of the current study and future prospects

The current study focused—as a proof of principle—on evaluating whether iPSC-derived neurons from one hESC-line exhibit similar DNA methylation patterns compared to isogenic hESC-derived neurons. A clear limitation of the current study is the lack of a profound DNA methylation analysis comparing pluripotent and differentiated cells, except for the *SNCA* locus. The current study did also not address the question whether certain CpG methylation patterns were erased or preserved during the reprogramming process which has to be determined using additional isogenic pluripotent, hESC- and iPSC-derived neural cells. Moreover, the complex issue of variation in genomic imprinting and X chromosome inactivation, too, would require more detailed analyses including, e.g., assessment of allele-specific expression, H3K27me accumulation, and *XIST* RNA levels, which are going beyond the scope of this study. Despite these limitations, our results suggest that the reprogramming process itself has no major impact on methylation patterns in iPSC-derived neurons. Yet, it remains to be determined how in vitro methylation patterns of hESC- and iPSC-derived neurons correlate to those of neurons derived from the native brain tissue. This is particularly relevant in the light of recent findings revealing substantial transcriptional differences between iPSC-derived and primary midbrain dopamine neurons [32].

## Conclusions

iPSC-derived neurons represent a valuable tool for modeling neurological disorders in vitro. While a number of studies have shown that the epigenetic status of iPSC closely mimics that of non-reprogrammed pluripotent stem cells (i.e., hESC), it had remained largely unclear whether neurons differentiated from iPSC adopt methylation patterns similar to those of neurons derived from non-reprogrammed cells. Using an isogenic system, this study demonstrates that ESC- and iPSC-derived neurons are highly similar with respect to DNA methylation and gene expression. While further studies are required to explore how DNA methylation in iPSC-derived neurons correlates to methylation patterns in primary neurons derived from the CNS tissue, our results suggest that the reprogramming process itself does not confound methylation patterns in iPSC-derived neurons. Thus, iPSC-derived neurons should represent a suitable tool for studying the impact of genetic variants on the epigenome of neurons.

## Methods

### Cell culture

HESCs (line I3) were maintained and differentiated to rosette-forming neural stem cells (NSCs) according to standard protocols and differentiated to neurons as described previously (Additional file 9) [14]. iPSCs were obtained and validated according to established protocols. In brief, NSCs were transduced with lentiviral TetON-system in order to generate a stable inducible cell line harboring the reprogramming factors OCT4 and KLF4 (pLVXTP-Tet-On (Clontech), FUW-OCT4, and FUW-KLF4 (Addgene)). Cells were induced to reprogram by the addition of 1 μg/ml doxycycline (Sigma-Aldrich) and cultured on irradiated mouse embryonic fibroblasts in Knockout Dulbecco’s modified Eagle’s medium containing 20% serum replacement, 1% non-essential amino acids, 1 mM L-glutamine, 0.1 mM ß-mercaptoethanol, and 4 ng/ml FGF2. Doxycycline was withdrawn upon colony formation, and doxycycline-independent colonies were mechanically isolated and further propagated. iPSC lines were differentiated to NSC and cultured as described [14]. Terminal differentiation of NSC was performed on Geltrex-coated dishes (Life technologies) in DMEM/F12 and Neurobasal (B27 supplement 1:50, penicillin/streptomycin 1:100, cAMP 300 ng/ml, and 1.6 g/l glucose) mixed at a 1:1 ratio. Neurons were cultured for 2, 4, and 6 weeks, and media were changed every second day (Additional file 9).

### Extraction of DNA and RNA

Genomic DNA was isolated using the DNeasy Blood & Tissue Kit (Qiagen) according to the manufacturer’s protocol. RNA was isolated using the RNeasy Mini Kit (Qiagen) according to the manufacturer’s protocol.

### cDNA synthesis and semiquantitative PCR

One microliter of total RNA was used for cDNA synthesis with the iScript cDNA Synthesis Kit (Bio-Rad Laboratories) according to the manufacturer’s instructions. A final concentration of 150 ng/μl of cDNA was used for real-time PCR with Power SYBR Green PCR Master Mix (Applied Biosystems) according to the manufacturer’s instructions. Possible contamination with genomic DNA was tested using samples processed without reverse transcriptase. Gene expression levels were normalized to human β-actin using the following primers (final concentration 10 μM): *SNCA* forward 5′-cgacgacagtgtggtgtaaag-3′ and *SNCA* reverse 5′-aaatgttggaggagcagtgg-3′.

### Bisulphite treatment and single CpG analysis

Bisulphite treatment of genomic DNA was performed with the EZ DNA Methylation-Gold Kit (Zymo Research) according to the manufacturer’s protocol. Amplicons were generated using region-specific primers (final concentration 10 μM each) with the recommended adaptors (Additional file 6). Purified PCR products were pooled in an equimolar ratio and sequenced after cluster formation on a MiSeq instrument benchtop sequencer with the sequencing-by-synthesis technology [16] according to the manufacturer’s protocol. Runs were set for “Generate FASTQ only” workflow in Illumina Experiment Manager. ESC and iPSC were analyzed as described elsewhere [33].

### Infinium HumanMethylation450 BeadChip

Genome-scale DNA methylation profiles were generated using Illumina’s Infinium HumanMethylation450 Beadchip assay (Illumina). The assay allows the determination of DNA methylation levels at > 450.000 CpG sites covering promoters and putative regulatory domains of all designable RefSeq genes. The Infinium Methylation Assay was performed according to the manufacturer’s instructions. The fluorescently stained chips were imaged using an Illumina HiScan scanner.

### Infinium HT12v4 BeadChip

Genome-wide gene expression profiles were generated using Illumina’s Infinium HT12v4 BeadChip assay (Illumina). The assay allows the determination of gene expression of > 47.000 probes covering designable RefSeq genes. The Infinium Expression Assay was performed according to the manufacturer’s instructions. The scanning was performed using an Illumina HiScan. Data from these images were loaded into Illumina’s GenomeStudio software suite (GS) and exported as a GS export file.

### Data analysis

Genome-wide methylation raw data were pre-processed using R statistics software suites minfi and RnBeads [34] to extract the data, subtract the background, and to normalize the data using internal controls present on the chips. Only CpGs with a detection *p* value < 0.01 in all samples were included (*n* = 484,932 of 485,577). All samples were analyzed as individual samples (*n* = 1). For genome-wide expression analysis, raw data was background subtracted and quantile normalized using R and the lumi software suite. Only probes with a detection *p* value < 0.01 in all samples were included (*n* = 20,586 of 39,050). All NSCs were analyzed as individual samples (*n* = 1); neuronal samples were analyzed in duplicates (*n* = 2). Further statistical analysis was performed with R statistics software or SPSS version 22. *p* values were based on a (paired) two-sided *t* test. Associations between variables (expression and DNA methylation) were measured by Pearson’s rank correlation.

For amplicon-based single gene CpG methylation analysis, sequencing reads obtained from the Illumina MiSeq were processed and assigned to the reference sequence and respective sample using specific tags in the universal portion of the adaptors. Methylation level and patterns were assessed using multiple sequence alignment using BiQ Analyzer HT [35]. All samples were analyzed as individual samples (*n* = 1) excluding all reads with a maximum fraction of unrecognized CpG sites of 10%. Data are shown as mean ± SD.

Probes identified in differential methylation/expression analyses were mapped to corresponding gene IDs according to the Illumina annotation. GO enrichment analysis was performed using the GOrilla tool (http://cbl-gorilla.cs.technion.ac.il/).

### Publicly available data

Published and pre-processed 450 K data from Fernandez-Santiago et al. [11] were downloaded from GEO accession number GSE51921. Non-publicly available data of non-dopaminergic neurons was kindly provided by R. Fernandez-Santiago. Published and pre-processed 450 K data from Kim et al. [15] were downloaded from GEO accession number GSE38217. For the comparison of ESC-derived and fibroblast-iPSC-derived neurons, we included all samples with a detection *p* value of < 0.01 and excluded all CpGs having some potential SNP bias (hES-NSC and hES-Neurons *n* = 4, ESC line H9, NPC line H9 and DAn line H9 *n* = 2, iPSC iPD and LRRK PD *n* = 2, iPSC control *n* = 3, DAn control and LRRK2 PD *n* = 4, DAn iPD *n* = 6, non-dopaminergic neurons control, LRRK2 PD and iPD *n* = 3). Principal component analysis was performed in R using the “prcomp” function. Obtained eigenvalues for principal components 1 and 2 were used for generation of PCA plot. The fibroblast-iPSC-derived non-dopaminergic neurons from the study of Fernández-Santiago et al. were cultured in DMEMF12/Neurobasal medium supplemented with 0.5xB27, 0.5xN2, and 2 mM GlutaMax and penicillin-streptomycin [11]. HESC- and iPSC-derived non-dopaminergic neurons from the isogenic stem cell model were cultured with slightly higher doses of N2 and B27 (1×) and in the presence of cyclic adenosine monophosphate (cAMP, 0.1×). Dopaminergic neurons (DAn) from the study of Fernández-Santiago et al. were generated using lentiviral transduction of LIM homeobox transcription factor alpha (LMX1A), co-cultured with a mouse with mouse PA6 feeding cells overexpressing sonic hedgehog (SHH) and cultured in the presence of fibroblast growth factor 8 (FGF8) [11]. Neurons were cultured for 30 days, and DAn exhibited a dopaminergic marker expression ranging from 44 to 67% (tyrosine hydroxylase (TH)/β-Tubulin III (TUJ1) ratio) [11]. Dopaminergic neural precursor cells from the study of Kim et al. (ESC line H9) were generated culturing hES colonies on irradiated PA6 or MA5 cells stably over-expressing SHH and cultured in insulin/transferrin/selenium/ascorbic acid/basic fibroblast growth factor (bFGF) medium [15]. Terminal differentiation of dopaminergic NSC into DAn was induced in the absence of bFGF but in the presence of brain-derived neurotrophic factor (BDNF), glial cell-derived neurotrophic factor (GDNF), and cAMP [15]. DAn were cultured for 42 days and exhibited a purity of 43% [15].

## Additional files

Additional file 1: A–C Interindividual analysis of DMCG comparing hES-Neurons to each iPSC-derived neuronal clone. D X chromosomal-based DMCG analysis using Fisher’s test. Methylation deltas were calculated as indicated and CpGs above applying a moderate (0.2) or high (0.5) threshold. (PDF 437 kb)

Additional file 2: A–D High correlation of technical replicates (hES-Neuron and iPS-Neuron clones 1–3) on the HT12v4 gene expression arrays. AU, arbitrary unit; Rep, replicate. E Highly similar expression profile comparing hES-NSC and iPS-NSC. AU, arbitrary unit. F Gene expression correlation profiles comparing hES-Neurons and iPS-Neurons. AU, arbitrary unit. (PDF 202 kb)

Additional file 3: Comparison of mean Pearson DNA methylation correlation coefficients. Comparisons were made including all ESC- and iPSC-derived NSC and neurons from the human isogenic stem cell system (isogenic hESC- and iPSC-derived NSC and neurons), all samples from the study of Fernández-Santiago et al. [11] except fibroblasts, and all samples from the study of Kim et al. [15]. iPSC, induced pluripotent stem cell; LRRK2, leucin-rich repeat kinase 2; PD, Parkinson’s disease; iPD, idiopathic PD; DAn, dopaminergic neuron; n, number; NSC, neural stem cell; hES(C), human embryonic stem (cell); I3, ESC-line I3; H9, ESC-line H9. (PDF 49 kb)

Additional file 4: Table of the top 20 differentially methylated CpGs (DMCG) in hESC- and iPSC-derived neurons (grouped together) compared to hESC- and iPSC-derived NSC (grouped together). Data was based on a paired *t* test (*p* < 0.001) applying a minimum methylation difference of 10%. Chr., chromosome. (PDF 47 kb)

Additional file 5: Mean DNA methylation levels of different genomic regions based on the genome-wide 450 K array data [AU ± SD]. AU, arbitrary unit; SD, standard deviation; hES, human embryonic stem cell; NSC, neural stem cell; iPS, induced pluripotent stem cell. (PDF 39 kb)

Additional file 6: Table of analyzed single genes and primers. (PDF 40 kb)

Additional file 7: Mean DNA methylation levels (%) of single genes (*APP*, *GNAS*, *MIR886*, *PMP22*, *SNCA* promoter, *SNCA* intron 1) of hESC-derived and iPSC-derived NSC and neurons. Analysis is based on individual samples. Data is shown as mean ± SD. (PDF 40 kb)

Additional file 8: DNA methylation levels of *SNCA* intron 1 in pluripotent stem cells (ESC line I3 and I6 (*n* = 1 each), iPSC line I3 (*n* = 3), ES- and iPS-NSC (line I3, *n* = 1 and *n* = 3, respectively), and ES- and iPS-Neurons (line I3, *n* = 1 and *n* = 3, respectively). ESC versus iPS-Neurons *p* = 0.03, iPSC vs. iPS-Neurons *p* = 0.02, and iPS-NSC vs. iPS-Neurons *p* = 0.02. (PDF 41 kb)

Additional file 9: A–C Immunocytochemical analysis of iPSC generated from hESC-derived NSC staining positive for the pluripotency-associated markers Oct4, Tra-1-60, and Tra-1-81. D–F Upon differentiation, these cells give rise to all three germ layers and express appropriate markers for endoderm (D; AFP), mesoderm (E, SMA), and ectoderm (F; TUBB3). G, H iPSC-derived NSC expressing the early neuroectodermal markers nestin, Dach1, ZO1, and PLZF. I, J 6-week-old iPSC-derived neurons staining for the neuronal marker MAP2 (I) and the neurotransmitter GABA (J). Nuclei are counterstained with DAPI. Scale bars: A–F, 75 μm; G–J, 10 μm. (PDF 3601 kb)
