# Supplementary material for: DNA methylation alterations in iPSC- and hESC-derived neurons: potential implications for neurological disease modeling
Source: Clin Epigenetics. 2018 Jan 29;10:13. doi: 10.1186/s13148-018-0440-0 (PMC5789607; doi:10.1186/s13148-018-0440-0)
Supplement: Supplementary file 1 — A–C Interindividual analysis of DMCG comparing hES-Neurons to each iPSC-derived neuronal clone. D X chromosomal-based DMCG analysis using Fisher’s test. Methylation deltas were calculated as indicated and CpGs above applying a moderate (0.2) or high (0.5) threshold. (PDF 437 kb) [file 13148_2018_440_MOESM1_ESM.pdf]

A

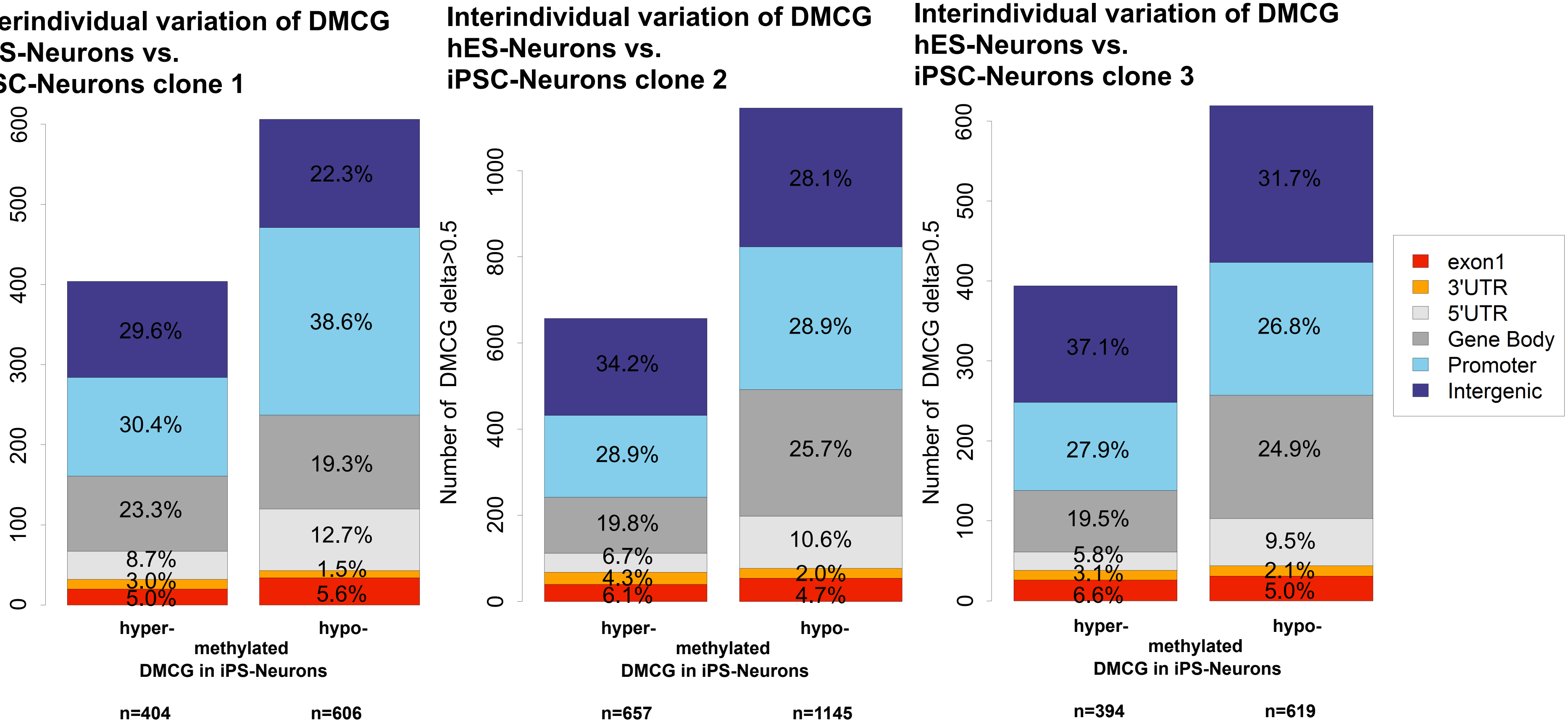

D X chromosomal DNA methylation variation analysis

Comparison (x_vs_y)	Number .x	Number .y	Delta.cut-off	Sig.x	Non-sig.x	sig.non.x	Non.sig.non.x	Fisher.test.p	Odds.ratio
hES-NSC vs. iPS-NSC	1	3	0.2	2148	11232	17857	473700	<2.2E-16	5.1
hES-Neurons vs. iPS-Neurons	1	3	0.2	2269	8963	18227	455473	<2.2E-16	6.3
hES-NSC vs. iPS-NSC	1	3	0.5	155	11232	734	473529	5.00E-84	8.9
hES-Neurons vs. iPS-Neurons	1	3	0.5	123	11109	566	484932	5.77E-70	9.5
