# Supplementary material for: DNA methylation alterations in iPSC- and hESC-derived neurons: potential implications for neurological disease modeling
Source: Clin Epigenetics. 2018 Jan 29;10:13. doi: 10.1186/s13148-018-0440-0 (PMC5789607; doi:10.1186/s13148-018-0440-0)
Supplement: Supplementary file 2 — A–D High correlation of technical replicates (hES-Neuron and iPS-Neuron clones 1–3) on the HT12v4 gene expression arrays. AU, arbitrary unit; Rep, replicate. E Highly similar expression profile comparing hES-NSC and iPS-NSC. AU, arbitrary unit. F Gene expression correlation profiles comparing hES-Neurons and iPS-Neurons. AU, arbitrary unit. (PDF 202 kb) [file 13148_2018_440_MOESM2_ESM.pdf]

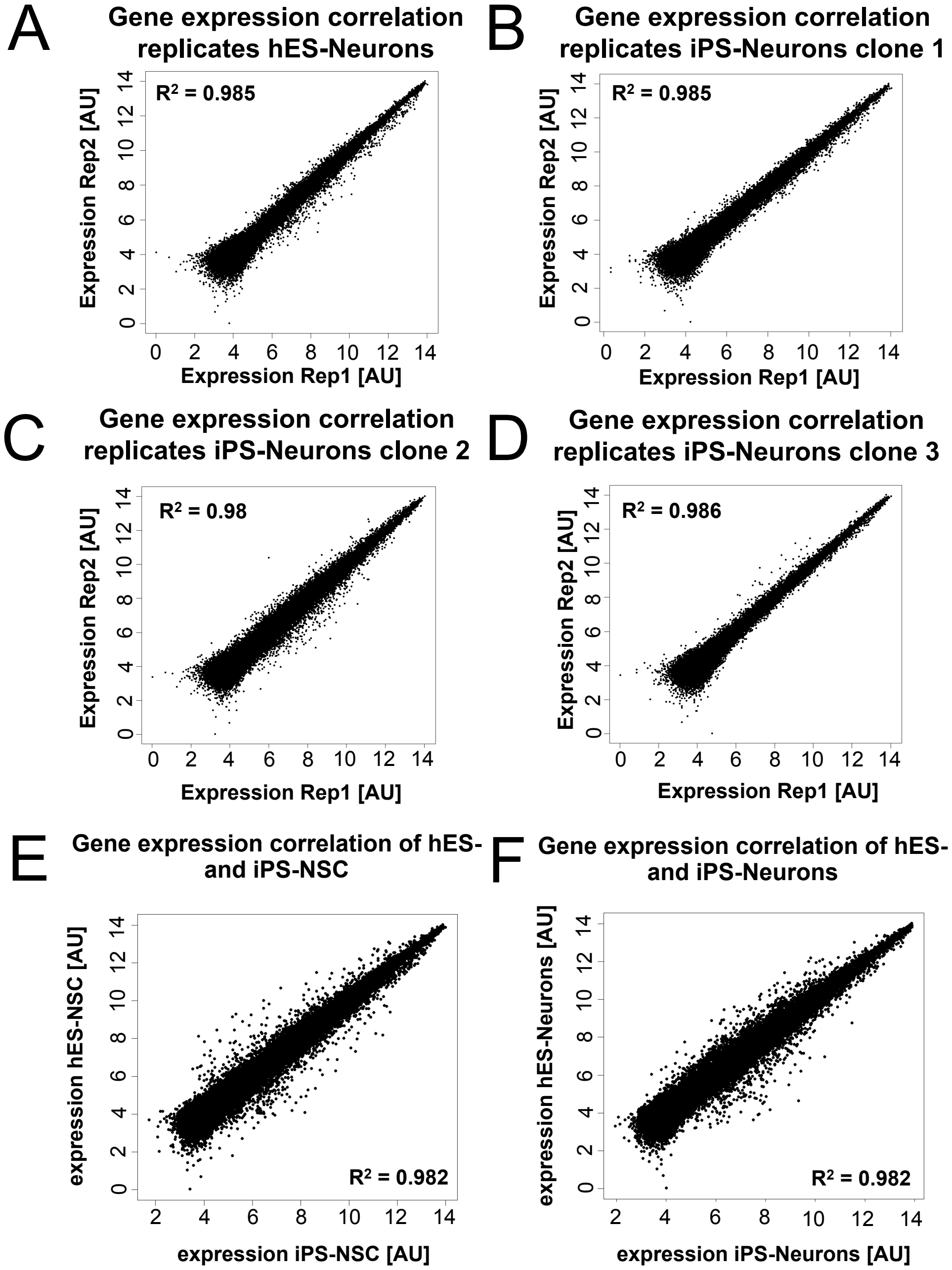
A
Gene expression correlation replicates hES-Neurons
R² = 0.985
Expression Rep2 [AU]
Expression Rep1 [AU]
B
Gene expression correlation replicates iPS-Neurons clone 1
R² = 0.985
Expression Rep2 [AU]
Expression Rep1 [AU]
C
Gene expression correlation replicates iPS-Neurons clone 2
R² = 0.98
Expression Rep2 [AU]
Expression Rep1 [AU]
D
Gene expression correlation replicates iPS-Neurons clone 3
R² = 0.986
Expression Rep2 [AU]
Expression Rep1 [AU]
E
Gene expression correlation of hES- and iPS-NSC
R² = 0.982
expression hES-NSC [AU]
expression iPS-NSC [AU]
F
Gene expression correlation of hES- and iPS-Neurons
R² = 0.982
expression hES-Neurons [AU]
expression iPS-Neurons [AU]
