# Supplementary material for: DNA methylation alterations in iPSC- and hESC-derived neurons: potential implications for neurological disease modeling
Source: Clin Epigenetics. 2018 Jan 29;10:13. doi: 10.1186/s13148-018-0440-0 (PMC5789607; doi:10.1186/s13148-018-0440-0)
Supplement: Supplementary file 3 — Comparison of mean Pearson DNA methylation correlation coefficients. Comparisons were made including all ESC- and iPSC-derived NSC and neurons from the human isogenic stem cell system (isogenic hESC- and iPSC-derived NSC and neurons), all samples from the study of Fernández-Santiago et al. [11] except fibroblasts, and all samples from the study of Kim et al. [15]. iPSC, induced pluripotent stem cell; LRRK2, leucin-rich repeat kinase 2; PD, Parkinson’s disease; iPD, idiopathic PD; DAn, dopaminergic neuron; n, number; NSC, neural stem cell; hES(C), human embryonic stem (cell); I3, ESC-line I3; H9, ESC-line H9. (PDF 49 kb) [file 13148_2018_440_MOESM3_ESM.pdf]

Mean genome-wide DNA methylation correlation										
		Fernandez-Santiago et al.								
		**Fibroblast-derived**								
		**iPSC control**	**iPSC LRRK2 PD**	**iPSC iPD**	**Neurons control (DAn)**	**Neurons LRRK2 PD (DAn)**	**Neurons iPD (DAn)**	**Neurons control**	**Neurons LRRK2 PD**	**Neurons iPD**
	number	n=2	n=2	n=3	n=4	n=4	n=6	n=3	n=3	n=3
de Boni et al.										
**hES-NSC (I3) and iPS-NSC (derived from I3 hES-NSC)**	n=4	0.84	0.83	0.84	0.91	0.91	0.91	0.91	0.91	0.91
**hES-Neurons (I3) and iPS-Neurons (derived from I3 hES-NSC)**	n=4	0.83	0.82	0.83	0.90	0.90	0.91	0.90	0.91	0.91
Kim et al.										
**ESC (H9)**	n=2	0.97	0.97	0.97	0.94	0.96	0.95	0.95	0.95	0.95
**hES-NSC (H9)**	n=2	0.88	0.87	0.88	0.91	0.92	0.92	0.93	0.94	0.93
**hES-Neurons (H9; DAn)**	n=2	0.89	0.87	0.89	0.92	0.93	0.93	0.94	0.95	0.94
