# Supplementary material for: DNA methylation alterations in iPSC- and hESC-derived neurons: potential implications for neurological disease modeling
Source: Clin Epigenetics. 2018 Jan 29;10:13. doi: 10.1186/s13148-018-0440-0 (PMC5789607; doi:10.1186/s13148-018-0440-0)
Supplement: Supplementary file 4 — Table of the top 20 differentially methylated CpGs (DMCG) in hESC- and iPSC-derived neurons (grouped together) compared to hESC- and iPSC-derived NSC (grouped together). Data was based on a paired t test (p < 0.001) applying a minimum methylation difference of 10%. Chr., chromosome. (PDF 47 kb) [file 13148_2018_440_MOESM4_ESM.pdf]

TOP 20 DMCG NSC VS. NEURONS					
**Probe ID**	**P value**	**Delta**	**Chr.**	**Position**	**Gene**
cg14059126	2.89E-07	0.2	2	235474894	n.a.
cg22248286	3.54E-07	0.4	2	236849184	AGAP1
cg18844163	4.86E-07	0.3	5	142362123	ARHGAP26
cg04700474	1.18E-07	0.1	6	110841946	n.a.
cg16353800	1.59E-07	0.5	18	72917943	ZADH2
cg00299839	3.61E-07	0.2	15	101609726	LRRK1
cg07270259	3.78E-07	0.4	10	24902536	PARD3
cg08059678	3.84E-07	0.1	16	70728425	VAC14
cg14691971	4.45E-07	0.4	8	102300350	n.a.
cg19924948	4.74E-07	0.4	2	11970722	n.a.
cg07093324	5.11E-07	0.5	2	114652143	ACTR3
cg06803850	5.17E-07	0.1	17	26926738	SPAG5
cg13501446	5.29E-07	0.9	9	138900624	n.a.
cg15033552	5.53E-07	0.4	1	223889141	CAPN2
cg19016972	6.38E-07	0.2	12	8012926	SLC2A14
cg14788673	6.45E-07	0.2	1	60164280	FGGY
cg15206981	6.50E-07	0.6	4	124323013	SPRY1
cg26373351	7.17E-07	0.5	12	12031304	ETV6
cg12081267	1.03E-07	0.4	2	98486185	TMEM131
cg18246610	1.04E-07	0.2	7	83671834	SEMA3A
