# Supplementary material for: DNA methylation alterations in iPSC- and hESC-derived neurons: potential implications for neurological disease modeling
Source: Clin Epigenetics. 2018 Jan 29;10:13. doi: 10.1186/s13148-018-0440-0 (PMC5789607; doi:10.1186/s13148-018-0440-0)
Supplement: Supplementary file 5 — Mean DNA methylation levels of different genomic regions based on the genome-wide 450 K array data [AU ± SD]. AU, arbitrary unit; SD, standard deviation; hES, human embryonic stem cell; NSC, neural stem cell; iPS, induced pluripotent stem cell. (PDF 39 kb) [file 13148_2018_440_MOESM5_ESM.pdf]

	MEAN METHYLATION						
	**All regions**	**Promoter**	**Gene Body**	**Exon1**	**3'UTR**	**5'UTR**	**Intergenic**
**hES-NSC**	0.53±0.35	0.34±0.33	0.54±0.34	0.53±0.35	0.54±0.34	0.54±0.35	0.53±0.35
**iPS-NSC clone 1**	0.54±0.34	0.35±0.33	0.54±0.34	0.54±0.34	0.54±0.34	0.54±0.34	0.54±0.34
**iPS-NSC clone 2**	0.53±0.35	0.34±0.33	0.53±0.35	0.53±0.35	0.53±0.35	0.53±0.35	0.52±0.35
**iPS-NSC clone 3**	0.53±0.34	0.34±0.33	0.53±0.34	0.53±0.34	0.53±0.33	0.53±0.34	0.52±0.34
**hES-Neuron**	0.54±0.34	0.35±0.33	0.54±0.34	0.54±0.35	0.54±0.34	0.54±0.34	0.54±0.34
**iPS-Neurons clone 1**	0.56±0.34	0.37±0.34	0.56±0.34	0.56±0.34	0.56±0.34	0.56±0.34	0.56±0.34
**iPS-Neurons clone 2**	0.55±0.34	0.36±0.33	0.55±0.34	0.54±0.34	0.55±0.34	0.55±0.34	0.55±0.34
**iPS-Neurons clone 3**	0.55±0.34	0.36±0.33	0.55±0.34	0.55±0.34	0.55±0.34	0.55±0.33	0.55±0.34
