# Supplementary material for: DNA methylation alterations in iPSC- and hESC-derived neurons: potential implications for neurological disease modeling
Source: Clin Epigenetics. 2018 Jan 29;10:13. doi: 10.1186/s13148-018-0440-0 (PMC5789607; doi:10.1186/s13148-018-0440-0)
Supplement: Supplementary file 6 — Table of analyzed single genes and primers. (PDF 40 kb) [file 13148_2018_440_MOESM6_ESM.pdf]

Gene	Number of CpGs	Forward primer	Reverse primer
**APP**	17	5’-GTTTTAAAGATAGAAATTTTAGGTTGT-3’	5’-AAACAATAAAAAAAAAAAATCTAAAACC-3’
**GNAS**	18	5’-GTGGGGTTAAAGGAGTTGATTGATT-3’	5’-CAAAAACAAAACCCCCTAAAAAATAA-3’
**MIR886**	20	5’-TTTTTAGTGGGTGGATTTTGTTTTT-3’	5’-AAAAATAACAAAACCTTCAAAATAACAA-3’
**PMP22**	4	5’-GGAAAGTAAATAAAGTTGGATATTGTT-3’	5’-ATCTCTCCTACAATCCTTTTCATTTA-3’
**SNCA intron 1**	20	5’-TATAAGGGTTGAGAGATTAGGT-3’	5’-TATAATAATTCTAATCCATCCAA-3’
**SNCA promoter**	17	5’-AAAATTTTGAAGATATTTGAATT-3’	5’-AAACTAAAAATAATACCTCTTCCT-3’
