# Supplementary figures and images for: DNA methylation alterations in iPSC- and hESC-derived neurons: potential implications for neurological disease modeling
Source: Clin Epigenetics. 2018 Jan 29;10:13. doi: 10.1186/s13148-018-0440-0 (PMC5789607; doi:10.1186/s13148-018-0440-0)

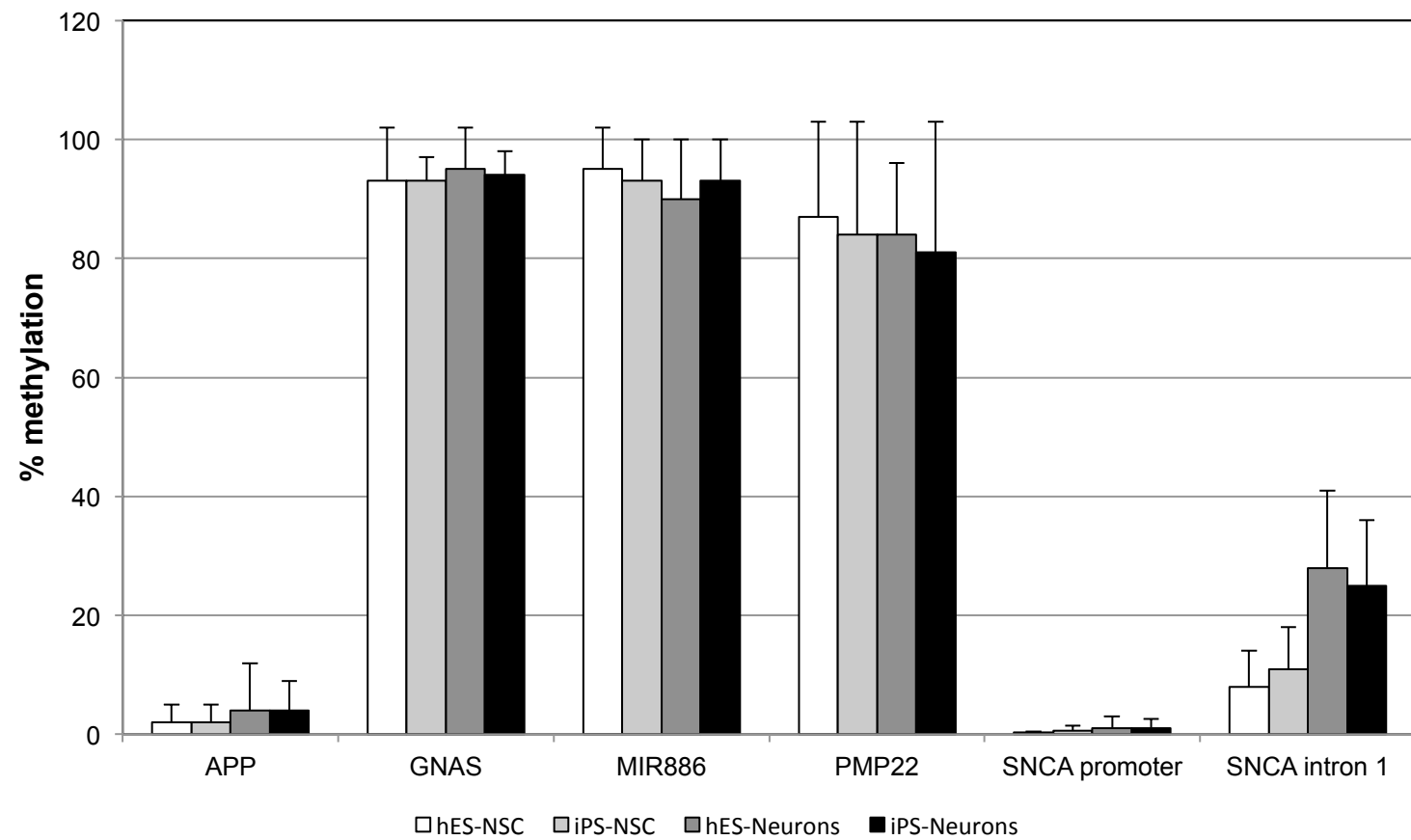

Supplement: Supplementary file 7 — Mean DNA methylation levels (%) of single genes (APP, GNAS, MIR886, PMP22, SNCA promoter, SNCA intron 1) of hESC-derived and iPSC-derived NSC and neurons. Analysis is based on individual samples. Data is shown as mean ± SD. (PDF 40 kb) [file 13148_2018_440_MOESM7_ESM.pdf]

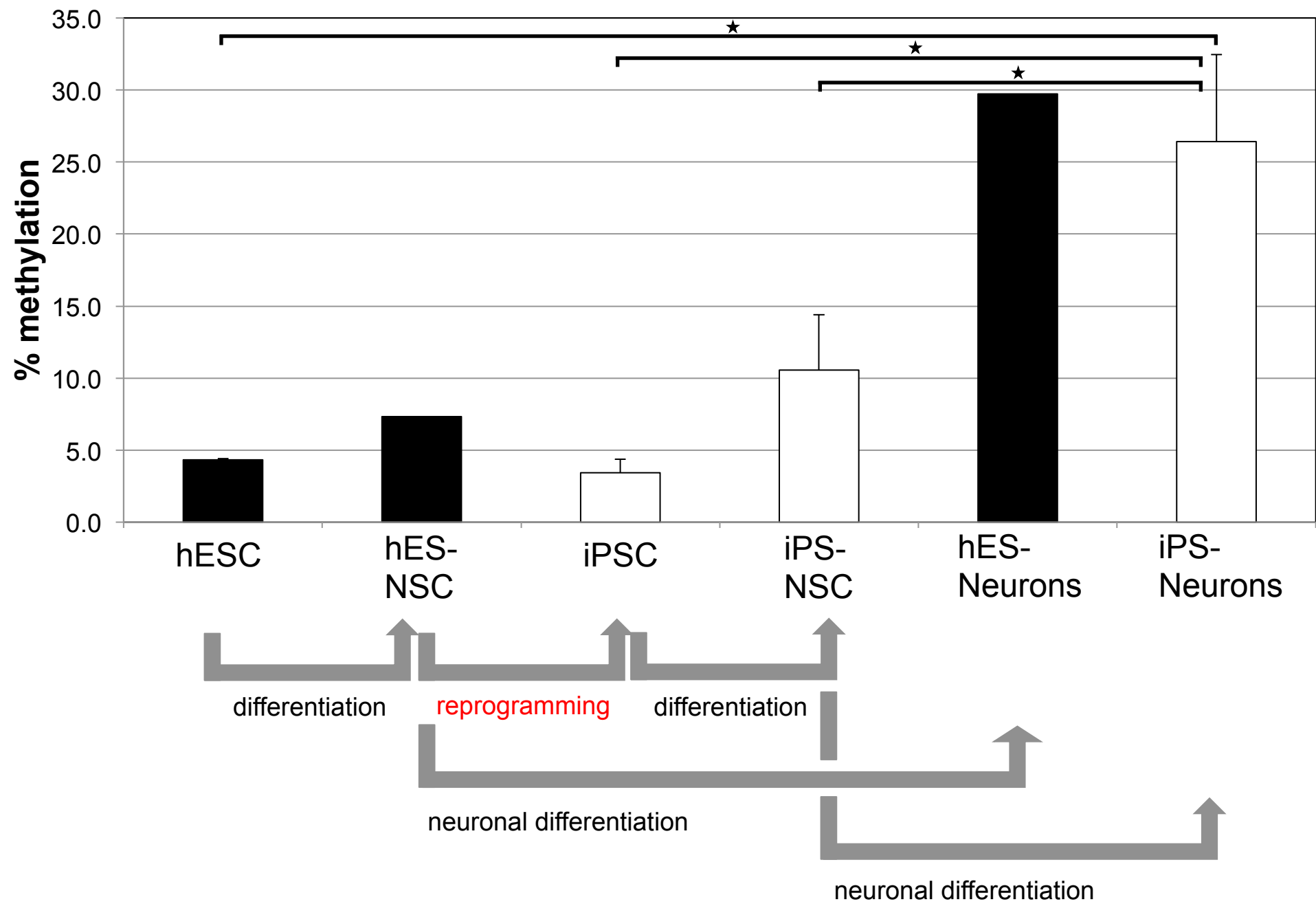

Supplement: Supplementary file 8 — DNA methylation levels of SNCA intron 1 in pluripotent stem cells (ESC line I3 and I6 (n = 1 each), iPSC line I3 (n = 3), ES- and iPS-NSC (line I3, n = 1 and n = 3, respectively), and ES- and iPS-Neurons (line I3, n = 1 and n = 3, respectively). ESC versus iPS-Neurons p = 0.03, iPSC vs. iPS-Neurons p = 0.02, and iPS-NSC vs. iPS-Neurons p = 0.02. (PDF 41 kb) [file 13148_2018_440_MOESM8_ESM.pdf]

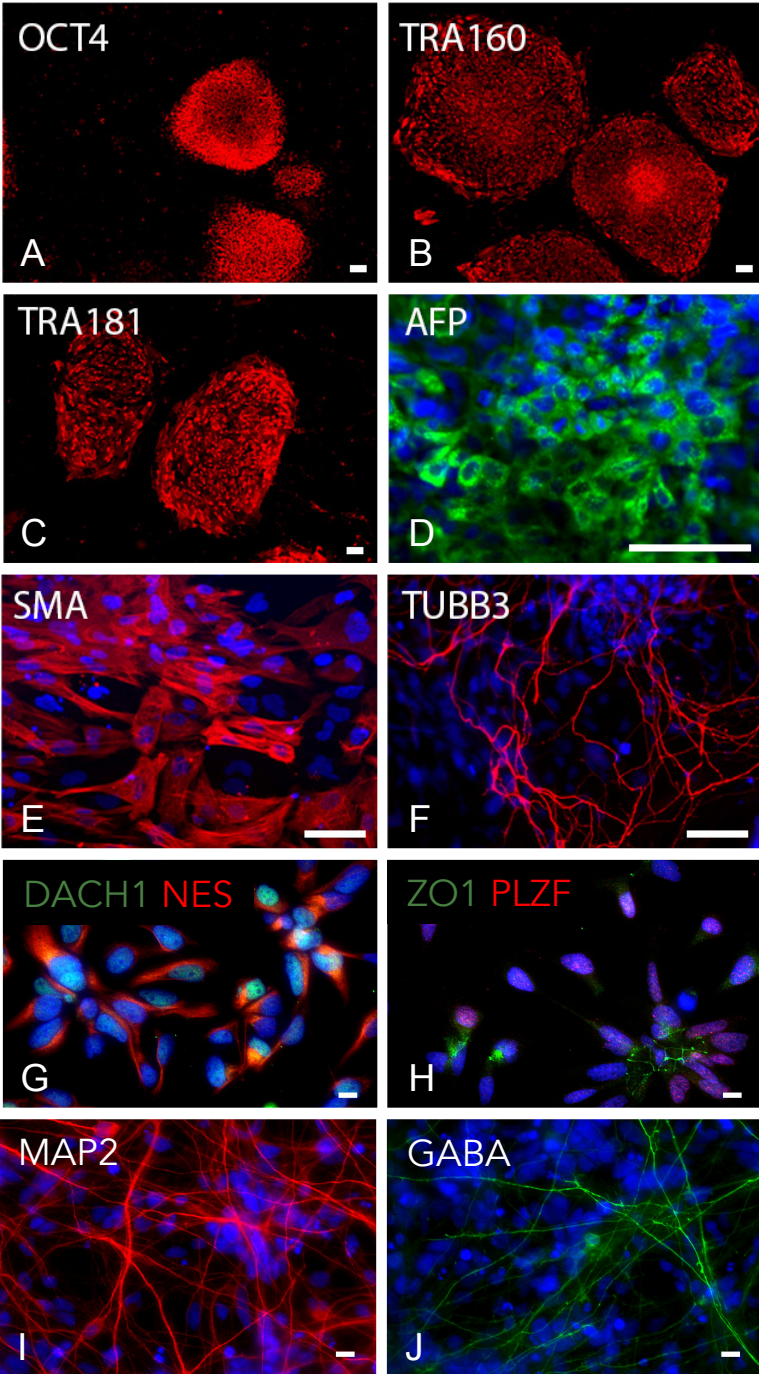

Supplement: Supplementary file 9 — A–C Immunocytochemical analysis of iPSC generated from hESC-derived NSC staining positive for the pluripotency-associated markers Oct4, Tra-1-60, and Tra-1-81. D–F Upon differentiation, these cells give rise to all three germ layers and express appropriate markers for endoderm (D; AFP), mesoderm (E, SMA), and ectoderm (F; TUBB3). G, H iPSC-derived NSC expressing the early neuroectodermal markers nestin, Dach1, ZO1, and PLZF. I, J 6-week-old iPSC-derived neurons staining for the neuronal marker MAP2 (I) and the neurotransmitter GABA (J). Nuclei are counterstained with DAPI. Scale bars: A–F, 75 μm; G–J, 10 μm. (PDF 3601 kb) [file 13148_2018_440_MOESM9_ESM.pdf]
